# Research on Quantitative Evaluation of Defects in Ferromagnetic Materials Based on Electromagnetic Non-Destructive Testing

**DOI:** 10.3390/s25113508

**Published:** 2025-06-02

**Authors:** Xiangyi Hu, Ruijie Xie, Ruotian Wang, Jiapeng Wang, Haichao Cai, Xiaoqiang Wang, Xiang Li, Qingzhu Guan, Jianhua Zhang

**Affiliations:** 1 School of Mechatronics Engineering, Henan University of Science and Technology, Luoyang 471003, China; 9906112@haust.edu.cn (R.X.); 13673978235@163.com (R.W.); 13233808128@163.com (J.W.); chc1226@haust.edu.cn (H.C.); wang_xq2002@163.com (X.W.); 2 International Education College, Henan University of Science and Technology, Luoyang 471000, China; 19837970301@163.com (X.L.); 231425090109@stu.haust.edu.cn (Q.G.); 3 Key Laboratory of High Efficiency and Clean Mechanical Manufacture, Ministry of Education of China, National Demonstration Center for Experimental Mechanical Engineering Education, School of Mechanical Engineering, Shandong University, Jinan 250061, China; jhzhang@sdu.edu.cn

**Keywords:** electromagnetic non-destructive testing, ferromagnetic material, quantitative evaluation, defect dimension, characteristic parameter

## Abstract

Defects are a direct cause of failure in ferromagnetic components, which can be evaluated via electromagnetic non-destructive testing (ENDT) methods. However, the existing studies exhibit several limitations (e.g., inaccurate quantification, over-reliance on algorithms, and non-intuitive result presentation, among others) in quantitative defect evaluation. To accurately describe the quantitative relationship between ENDT signals and defect dimensional parameters, the electromagnetic model and electromagnetic induction model are introduced in this paper to elucidate the physical mechanism of ENDT, as both models provide a basis for the selection of the constitutive relationship for simulation analysis. Then, a higher precision three-dimensional nonlinear finite element simulation model is established, and the effects of the excitation parameters and detection positions on signal characteristics are investigated. The simulation results indicate that the excitation frequency influences both the detection depth and sensitivity of ENDT, while the voltage amplitude affects the peak strength of the magnetic signal. Consequently, the excitation parameters are determined to be a 10 Hz frequency with a 25 V amplitude. Based on the characterization of signal peaks at positions of 0°, 90°, 180°, and 270°, the characteristic parameter *K_A_* of the peak electrical signal curve is proposed as a quantitative index for evaluating defects. The quantitative experimental results show that *K_A_* is related to the defect dimension. Specifically, the *K_A_* value monotonically decreases from a constant greater than 1 to a constant less than 1 as the defect length increases, *K_A_* is positively correlated with the defect width, and *K_A_* follows a parabolic trend (first increase and then decrease) as the defect depth increases. Notably, *K_A_* values associated with defect width and depth remain below 1. All the above findings provide a basis for evaluating defect dimensions. The results of this paper provide a novel ENDT method for evaluating defects, which is of great significance for improving the accuracy of ENDT and promoting its engineering applications.

## 1. Introduction

Ferromagnetic components play a crucial role in modern industry applications, being widely used in aerospace equipment, manufacturing machinery, infrastructure, and the petroleum and petrochemical industry. Statistical data indicate that annual global direct economic losses from metallic component failures exceed USD 700 billion [1], of which over 60% can be reduced or avoided by inspection techniques and life assessment. Crack defects are a direct factor contributing to the failure of ferromagnetic components, which typically originate from the accumulation and propagation of microcracks that develop within the material under service loads [2,3]. Consequently, the quantitative evaluation of defects in ferromagnetic materials presents a significant challenge in the field of science and engineering. ENDT can realize the quantitative detection of defects according to the magnetic signal variations induced by defects, which is of great significance for damage quantitative evaluation in ferromagnetic components.

Over recent decades, ENDT has obtained a series of research results [4,5,6,7,8,9,10,11,12,13,14], with a systematic framework for defect assessment having been established based on the eddy current method [4,5], alternating magnetic field method [6,7], magnetic flux leakage method [8,9,10,11], magnetic memory method [12,13,14], and so on. Currently, ENDT for defect characterization continues to attract the attention of many scholars, and a series of valuable research results have been achieved. For example, Hwang et al. [15] investigated the magnetic field distribution surrounding a rectangular slot in ferromagnetic components, validating the efficacy of ENDT for defect quantitative characterization. Dutta et al. [16] developed a magnetic dipole model for analyzing defective magnetic flux leakage signals, emphasizing the critical role of the tangential component signal in defect detection. Xu et al. [17] used a multi-frequency, exciting three-dimensional alternating current magnetic field detection system to evaluate defects in high-speed rails, thereby establishing a quantitative model capable of characterizing defects with depths spanning 2 mm-8 mm and surface angles ranging from 30° to 75°. Wang et al. [18] proposed a time-domain analysis method for alternating current leakage flux signals, enabling the classification and quantification of internal/external defects in ferromagnetic materials. Yuan et al. [19] found that the Bayesian network processing of the integrated Bx and Bz signals enhances crack defect quantification accuracy in alternating current electromagnetic fields. Ru et al. [20] designed an innovative multi-parameter electromagnetic sensor by integrating the alternating current field method with the magnetic flux leakage method, realizing the identification of different types of pipeline defects. Guo et al. [21] combined the electromagnetic acoustic method with the pulsed eddy current method, achieving hybrid defect detection capabilities. Grochowalski et al. [22] constructed a three-dimensional pulsed multifrequency excitation sensor model, employing k-Nearest Neighbors algorithms for defect parameter determination via spectral analysis. While these methods exhibit distinct advantages, a novel ENDT demonstrates superior environmental adaptability and detection reliability for practical applications [14]. Therefore, this study focuses on the novel ENDT for defect evaluation. There are two forms of ENDT detection probes. The first type of detection probe is constructed by placing an induction coil or Hall element capable of acquiring magnetic signals in the middle of a U-shaped pole wrapped around an excitation coil [23,24,25]. The other type of detection probe consists of a U-shaped magnetic core with excitation and induction coils wound around its two poles. Defects within the specimen induce magnetic signal distortion, resulting in variations in magnetic flux through the induction coil [26]. Compared with the first type, the second type of detection probe features a more compact structure, simpler manufacturing process, and enhanced capability for detecting deep-seated defects. Therefore, this study adopts the second type of probe for detection.

Conventional ENDT for defect evaluation frequently encounters significant signal interference under complex operational environments, necessitating the integration of intelligent algorithms for enhanced data processing. For instance, Liu et al. [27] developed a cascaded attention feature fusion network to improve the quantitative accuracy of ENDT for defects. Zhang et al. [28] created the Chaos Opposition Group Learning–Marine Predators Algorithm–Extreme Gradient Boosting framework, validating its superior performance through comparative experiments with three machine learning models. Sun et al. [29] implemented a physics-informed doubly fed cross-residual network for defect detection signal analysis, achieving high-precision quantification of defect dimensions. The current research predominantly emphasizes the intelligent processing of detection data, where defect evaluation accuracy is inherently dependent on algorithmic precision. On the whole, the evaluation accuracy of defects comes from detection accuracy and algorithm recognition accuracy; however, it cannot intuitively or quickly reflect the characteristics of defects, which is not conducive to the engineering application of ENDT. Moreover, high-precision identification algorithms require a large number of detection data points. Therefore, it is necessary to investigate a new method that can intuitively and accurately evaluate defects with only a small number of data points.

To enhance the practicality and applicability of ENDT for defect quantification, a simplified electromagnetic induction model was introduced in this study to elucidate the physical mechanism of ENDT. Based on the theoretical model, a high-precision, three-dimensional nonlinear finite element simulation model was established. The evolution of magnetic/electric signals during the ferromagnetic material detection process was systematically investigated, and a novel signal processing method named the characteristic parameter was proposed. The quantitative relationship between defect lengths, defect widths, detection depths, and detection signal is investigated through experiments. The results of this paper lay the foundation for high-accuracy defect characterization by ENDT techniques.

## 2. Theoretical Framework

Under the excitation of an alternating electromagnetic field, defects in ferromagnetic specimens will affect the distribution of magnetic lines of force, resulting in a change in the detection signal. According to the characteristics of the ENDT of defects, the simulation analysis should include an electromagnetic field module and a circuit module. This section introduces the electromagnetic model and electromagnetic induction model involved in ENDT, which provide theoretical support for the quantitative ENDT of defects.

### 2.1. Electromagnetic Model

**Figure 1:**
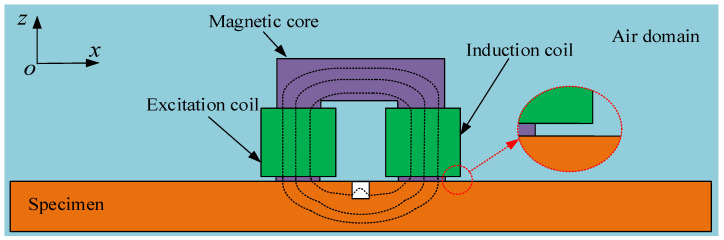
Schematic diagram of ENDT of ferromagnetic specimens containing defects.

A simplified model of ENDT is shown in Figure 1. The detection probe consists of a magnetic core, an excitation coil, and a detection coil. To simplify the analysis process, both the specimen materials and magnetic core materials are regarded as homogeneous and isotropic linear materials. A sinusoidal electrical signal *I_alter_* = *I*_0_*sin*(*ω_alter_t*) with angular frequency *ω_alter_* needs to be applied to the excitation coil during the detection process, which will generate an alternating magnetic field in the core. The magnetic field within the coil’s magnetic core is assumed to be uniform. The magnetic field enters the specimen from the magnetic core–specimen contact surface, after which it passes through the core inside the induction coil and generates an induced current in the induction coil. When a defect is present in the specimen, it will lead to a change in the distribution of magnetic inductance, which in turn will cause a variation in the magnetic flux passing through the induction coil. The propagation of the alternating electromagnetic field throughout the detection process satisfies Maxwell’s equations [30,31]:

(1) $$\nabla \times E=-\frac{\partial B}{\partial t}$$

(2) $$\nabla \times H=J+\frac{\partial D}{\partial t}$$

(3) ∇⋅B=0

(4) ∇⋅D=ρ

where ∇ is a differential operator, ***E*** is the electric field strength, ***B*** is the magnetic flux density, ***H*** is the magnetic field strength, ***J*** is the current density, ***D*** is the electric flux density, and *ρ* is the charge density.

The skinning depth of the electric and magnetic fields along the surface to be measured during sinusoidal alternating excitation can be expressed by *δ_skin_*:

(5) $$\delta_{\mathrm{skin}}=\frac{1}{\sqrt{{\pi f}_{\mathrm{alter}}\mu_{0}\mu_{r}\sigma_{\mathrm{ec}}}}$$

*ω_alter_* = 2π*f_alter_* (6)

where *f_alter_* is the frequency of the excitation electrical signal, *σ_ec_* is the conductivity of the material, and *μ_r_* is the relative magnetic permeability of the sample to be tested.

Inside the specimen convergent skin layer *δ_skin_*, the magnetic field obeys the relationship $\nabla^{2}H=k_{\mathrm{alt}}^{2}H$, where $k_{alt}^{2}=i\omega_{alter}\mu_{0}\mu_{r}\sigma_{ec}$ [30]. According to the relationship between the magnetic flux density ***B*** and the magnetic vector potential ***A***, ***B*** = ∇ × ***A***. Assuming that the material conforms to a linear law, the magnetic flux density ***B*** and the magnetic field satisfy ***B*** = *μ*_0_ *μ_r_ **H***. The magnetic field can be expressed as ***H*** = ***v****_r_*∇ × ***A***, where ***v****_r_* is the magneto-resistivity of the material, and the relationship between ***v****_r_* and *μ_r_* is ***v****_r_* = 1/*μ_r_*. When an alternating current is applied to the excitation coil, the spatial electromagnetic field in the vicinity of the ideal specimen (no defects or localized stress concentration areas) is parallel to the surface of the specimen and shows a uniform distribution. Combined with the principle of electromagnetic induction, the electromagnetic field vector potential function in the air domain during the detection process satisfies the Laplace equation:

(7) $$\frac{\partial^{2}A_{\mathrm{alter}}\left(x,y,z\right)}{\partial x^{2}}+\frac{\partial^{2}A_{\mathrm{alter}}\left(x,y,z\right)}{\partial y^{2}}+\frac{\partial^{2}A_{\mathrm{alter}}\left(x,y,z\right)}{\partial z^{2}}=0$$

where ***A****_alter_* (*x*, *y*, *z*) is the vector potential function of the electromagnetic field.

When there is no defect in the specimen, the electromagnetic field boundary condition at its surface (*z* = 0) can be written as [31]

(8) $$\frac{\partial^{2}A_{\mathrm{alter}}\left(x,y,z\right)}{\partial x^{2}}+\frac{\partial^{2}A_{\mathrm{alter}}\left(x,y,z\right)}{\partial y^{2}}+\frac{k_{\mathrm{alter}}}{\mu_{r}}\frac{\partial A_{\mathrm{alter}}\left(x,y,z\right)}{\partial z}=0$$

When there is a defect of width *d_ws_* in the specimen, according to the Lewis boundary condition, the electromagnetic field boundary condition can be written as

(9) $$\frac{\partial^{2}A_{\mathrm{alter}1}\left(x,y,z\right)}{\partial x^{2}}+\frac{\partial^{2}A_{\mathrm{alter}1}\left(x,y,z\right)}{\partial y^{2}}+\frac{k_{\mathrm{alter}}}{\mu_{r}}\frac{\partial A_{\mathrm{alter}1}\left(x,y,z\right)}{\partial z}=-\left(2+\frac{d_{\mathrm{wid}}k_{\mathrm{alter}}}{\mu_{r}}\right)H_{z0}\left(x,0\right)\delta \left(y\right)$$

where *δ*(*y*) is the unit pulse function. ***H****_z_*_0_ (*x*,0) = ***H****_z_* (*x*, *z*)|*_z_*_=0_ is the component of the magnetic field perpendicular to the crack opening. When the crack is a stress corrosion crack, *d_ws_* = 0.

Notably, Equation (7) represents the electromagnetic boundary conditions in the air domain, while Equations (8) and (9) correspond to the material-adjacent regions. Specifically, Equations (8) and (9) show the boundary conditions for defect-free and defect samples to be tested, respectively. According to the change law of spatial electromagnetic fields, the vector potential function around the defect of the test sample satisfies ***A****_total_* = ***A****_alter_* + ***A****_alter_*_1_, from which the analytical solution of the electromagnetic field near the defect of the sample can be obtained, and the value of the electromagnetic field distortion near the defect is obtained.

### 2.2. Electromagnetic Induction Model

During the magnetization process, the magnetic core, sample, and air gap between the magnetic core and sample collectively form a closed magnetic circuit, as shown in Figure 1. When an alternating current is applied to the excitation coil, a magnetomotive force is generated in the core, *F_m_* = *N_coil_I_coil_* (where *F_m_* is the magnetomotive force generated by the excitation coil, *N_coil_* is the number of turns of the excitation coil, and *I_coil_* is the excitation current). This induces an alternating magnetic flux Փ*_m_* through the circuit. According to Ohm’s law for magnetic circuits, the magnetic flux Փ*_m_* can be expressed as $\Phi_{m}=F_{m}/R_{m}$ (where Փ*_m_* is the total magnetic flux generated by the excitation coil, and *R_m_* is the total magnetoresistance of the circuit) [32]. When the magnetic flux leakage of the core magnetic circuit is disregarded, the total magnetic flux of the entire magnetic circuit can be expressed as

(10) $$\Phi_{m}=\Phi_{s}+\Phi_{d}=\frac{F_{m}}{R_{m}}$$

where Փ*_s_* is the magnetic flux through the magnetized region of the sample, and Փ*_d_* is the magnetic flux leakage generated at the defect.

When the magnetic core, air gap, sample, and defect in the magnetic circuit are all uniform media with consistent dimensions (i.e., each medium maintains identical geometry and magnetic reluctance within its respective magnetic path). The total reluctance of the entire magnetic circuit comprises the core reluctance *R_c_*, air gap reluctance *R_a_*, material reluctance *R_t_*, and defect resistance *R_k_* [32]. The total magnetic reluctance can be expressed as

(11) $$R_{m}=R_{c}+2R_{a}+\frac{R_{t}R_{k}}{R_{t}+R_{k}}=\frac{l_{c}}{\mu_{c}S_{c}}+\frac{2l_{a}}{\mu_{a}S_{a}}+\frac{\frac{l_{t}}{\mu_{t}S_{t}}\frac{l_{k}}{\mu_{k}S_{k}}}{\frac{l_{t}}{\mu_{t}S_{t}}+\frac{l_{k}}{\mu_{k}S_{k}}}=\frac{l_{c}}{\mu_{c}S_{c}}+\frac{2l_{a}}{\mu_{a}S_{a}}+\frac{l_{t}l_{k}}{l_{t}\mu_{k}S_{k}+l_{k}\mu_{t}S_{t}}$$

where *l_c_*, *l_a_*, *l_t_*, and *l_k_* are the effective magnetic path equivalent lengths in the core, air gap, sample, and defect, respectively. *μ_c_*, *μ_a_*, *μ_t_*, and *μ_k_* are the equivalent magnetic permeabilities of the core, air, sample, and defect, respectively. *S_c_*, *S_a_*, *S_t_*, and *S_k_* are the effective cross-sectional equivalent areas of the magnetic circuit in the core, air gap, sample, and defect, respectively.

Therefore, the leakage flux generated at the defect can be expressed as

(12) $$\Phi_{d}=\frac{F_{m}R_{t}}{\left(R_{c}+2R_{a}\right)\left(R_{t}+R_{k}\right)+R_{t}R_{k}}$$

From Equations (11) and (12), it can be seen that a variation in defect dimension alters the magnetic circuit reluctance, thereby modifying the flux leakage signal. Furthermore, these changes induce a corresponding variation in the magnetic flux through the core of the induction coil. The relationship between the magnetic flux Փ*_in_* through the core inside the induction coil and the magnetic flux density *B_c_* is given by [32] $\Phi_{in}=B_{c}S_{c}$ (where *S_c_* is the effective cross-sectional area of the core’s magnetic circuit, and *B_c_* is the magnetic flux density within the induction coil core). According to electromagnetic induction theory, the induced current in the detection coil can be expressed as $I_{out}=\frac{U_{in}}{R_{total}}=-\frac{N_{in}}{R_{total}}\frac{d\Phi_{in}}{dt}=-\frac{N_{in}S_{c}}{R_{total}}\frac{dB_{c}}{dt}$ (where *N_in_* is the number of turns of the detection coil, and *R_total_* is the total resistance of the entire circuit, with a constant value).

The defects in samples alter the magnetic field distributions, which can be numerically resolved through finite element analysis by applying the boundary conditions defined in Equation (9). In closed magnetic circuits, defect-induced flux leakage reduces effective magnetic flux penetration through the induction coil’s core. The above changes are ultimately changes in the output electric signal of the induction coil, thus realizing the effective detection and characterization of defects in ferromagnetic materials.

## 3. Simulation Analysis

To more accurately characterize the electromagnetic detection process of defects, this study develops a three-dimensional simulation model that replicates actual testing conditions. This model eliminates the conventional simplifications of linear material properties and identical cross-sections along the detection path while preserving the isotropic nature assumption to balance physical accuracy with computational feasibility. The model incorporates experimentally measured magnetization curves of the test specimen to precisely describe the material’s nonlinear magnetic properties. Subsequent investigations examine the influence of excitation parameters and defect dimensions on detection signals.

### 3.1. Simulation Model and Simulation Parameters

The Q345R steel (U.S. SA516-Gr.70 steel) used in this paper is a classical boiler vessel material, which is a hot-rolled, medium-thickness steel for boiler and pressure vessel applications, produced by the medium-thickness plate division of Liaoning Anshan Iron & Steel Co., Ltd. (Grade: Q345R, Melting No.: 19AD0844, Batch No.: 2590710400, Execution Standard: GB/T 713-2014). Its chemical composition is shown in Table 1.

**Figure 2:**
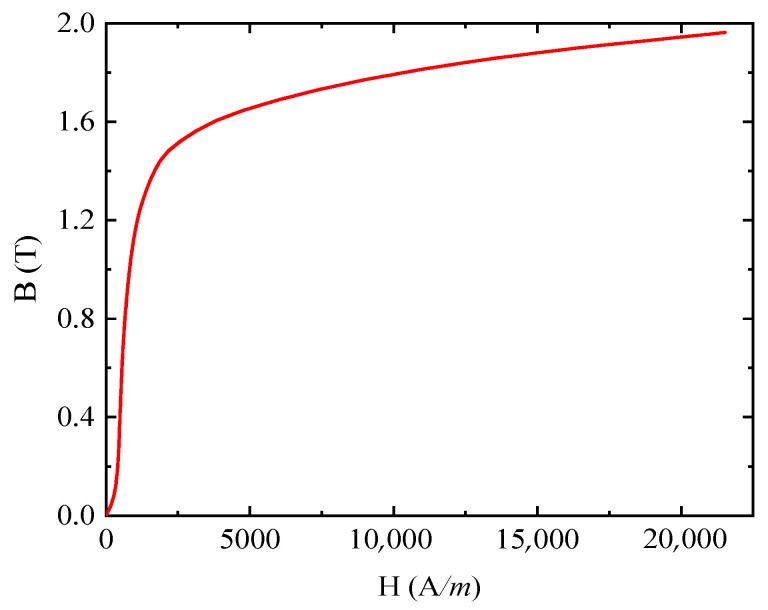
The magnetization curves of the Q345R sample.

Q345R steel was processed into 100 mm × 6 mm × 2 mm block samples with the wire-cut electrical-discharge machining technique (WEDM), and the machined surfaces of the Q345R samples were polished with 320-mesh, 600-mesh, 800-mesh, and 1000-mesh metallographic sandpaper, respectively. After the sample was polished to be smooth and scratch-free, it was ultrasonically cleaned with anhydrous ethanol. After that, the sample was placed in a muffle furnace to be heated up to 500 °C at a rate of 20 °C/min and then cooled down in a furnace after 2.5 h of the maintenance period for stress relief annealing. Finally, the Q345R samples were measured with the FE-2100SD ferromagnetic material measuring system according to GB/T 13012-2008 and GJB 937-90 standards, and the magnetization curve obtained is shown in Figure 2.

A model simulating the real environment of ENDT was built to study the variation of magnetic/electrical signals in the defect-containing specimen. The length × width × height of the specimen was 150 mm × 50 mm × 6 mm. The cross-section of the U-shaped core was a square with a side length of 4 mm, and the total length × width × height was 16 mm × 4 mm × 16 mm. The inner diameter, outer diameter, and height of the coil wound on the U-shaped core were 5 mm, 6.5 mm, and 10 mm, respectively. The entire solution domain was represented by a cube with a length × width × height of 420 mm × 225 mm × 275 mm. Other parameters of the simulation model are shown in Table 2.

**Figure 3:**
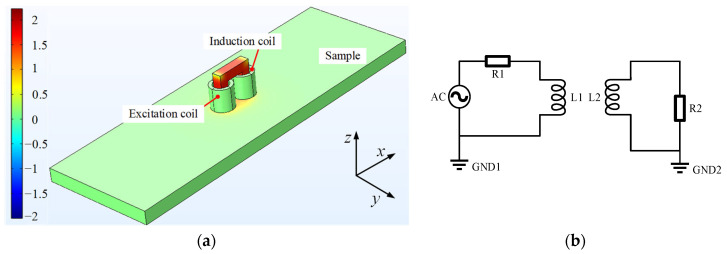
Simulation model and equivalent circuit of ENDT. (**a**) The simulation model. (**b**) The equivalent circuit.

The simulation model is shown in Figure 3a. In the electromagnetic field analysis module, the hysteresis effect was neglected. The B-H curve shown in Figure 2 was selected as the electromagnetic constitutive model of the specimen, which can accurately describe the nonlinear magnetic properties of the material. It is worth noting that this differs from the assumption that linear materials are used in Section 2. The electromagnetic constitutive model of the air domain is *B* = *μ*_0_*μ_r_H*, and the magnetic core satisfies the Jiles–Atherton (J-A) model with the parameters shown in Table 3 [33]. In meshing, tetrahedral cells are used throughout the finite element region. The coil excitation is driven by the circuit, and the connections between the components are shown in Figure 3b.

### 3.2. Determination of Excitation Parameters

(1) The influence of excitation signal frequency on magnetic flux density

**Figure 4:**
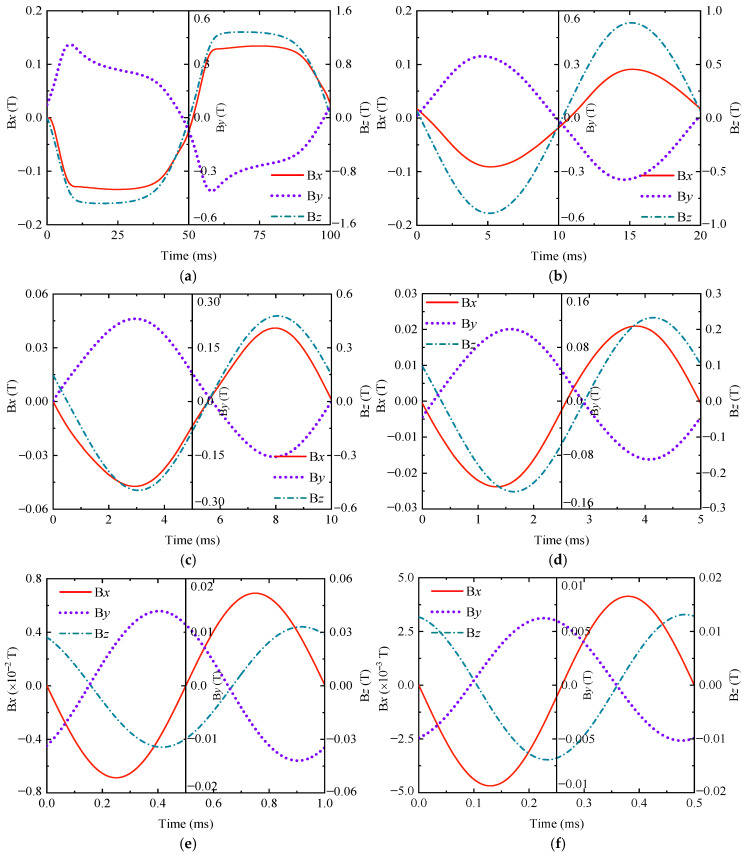
The magnetic flux density signals at different frequencies. (**a**) 10 Hz; (**b**) 50 Hz; (**c**) 100 Hz; (**d**) 200 Hz; (**e**) 1000 Hz; and (**f**) 2000 Hz.

A sinusoidal excitation signal with an amplitude of 25 V and frequencies of 10 Hz, 50 Hz, 100 Hz, 200 Hz, 1000 Hz, and 2000 Hz was used for the simulation and analysis of ENDT. In the post-processing module of the finite element software Comsol 6.1, the magnetic flux density signals through the core during steady-state operation at each frequency were extracted, as shown in Figure 4. At low excitation frequencies, the B*x* and B*z* components of the magnetic flux density signal share identical phases, whereas B*y* exhibits a phase in opposition to both. As frequency increases, B*z* progressively lags B*x*, with their phase difference amplifying. Since the signal period is frequency-dependent, instead of the period length being chosen as the electromagnetic field comparison parameter, the peak value of the signal is often taken as the comparison feature.

**Figure 5:**
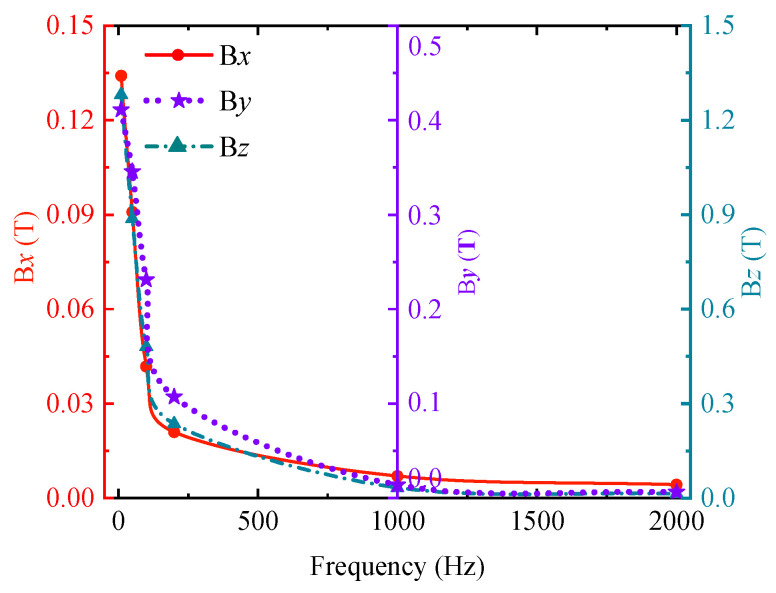
Variation in peak magnetic flux density signals at different frequencies.

Figure 5 shows the variation of the peak value of the magnetic flux density signal with the excitation frequency. From the diagram, it can be seen that the peak values of Bx, By, and Bz decrease nonlinearly with the increase of frequency, and the decrease rate also decreases gradually, so that the peak value of the magnetic flux density signal finally tends to a stable value. When the excitation frequency is large, the magnetic signal is small, the skin depth is also small, and the defect and stress state at the deeper position cannot be detected. The magnetic signal disturbance caused by surface defects and elastic–plastic deformation is also relatively small. It is not easy to separate the disturbance signal of ENDT. At this time, it is often necessary to enhance the strength of the excitation source. When the excitation frequency is small, the skin depth is relatively large. For thin plate samples, the magnetic field can easily break through the sample, resulting in inaccurate test results. Therefore, the change in excitation source frequency not only affects the effective detection range of ENDT but also affects the sensitivity of ENDT.

(2) The influence of excitation signal amplitude on magnetic flux density

**Figure 6:**
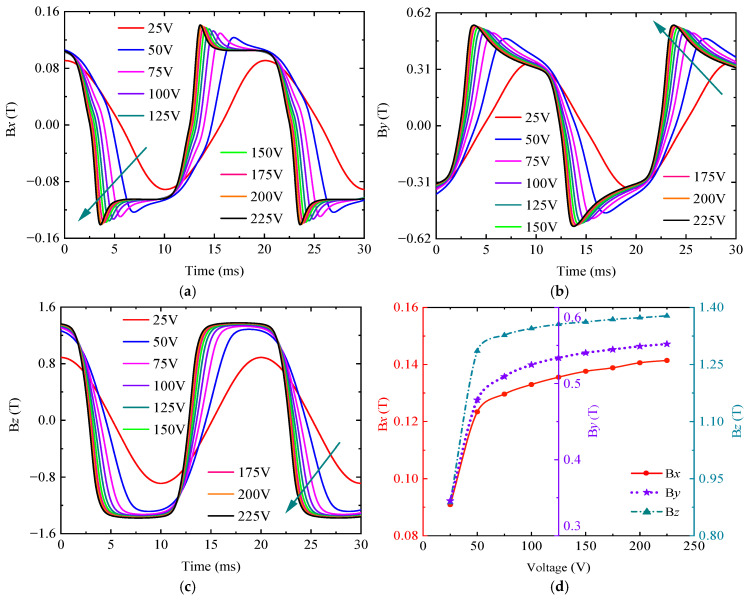
Magnetic flux density signals under different excitation voltages. (**a**) *Bx*. (**b**) *By*. (**c**) *Bz*. (**d**) Peak value of magnetic flux density signal.

The intensity of the excitation source determines the intensity of the detection signal. To effectively separate the disturbance signal of the damage, it is necessary to select the appropriate excitation source intensity. The sinusoidal excitation signals with excitation frequencies of 50 Hz and amplitudes of 25 V, 50 V, 75 V, 100 V, 125 V, 150 V, 175 V, 200 V, and 225 V were used for the simulation, and the results are shown in Figure 6.

As shown in Figure 6, with the increase in the excitation signal intensity, the shape of the magnetic flux density signal curve is obviously distorted, and the signal peak also increases nonlinearly. However, its increase rate gradually decreases, showing that the signal peak first increases rapidly and then increases slowly and gradually tends to be stable. The reason for this phenomenon is that the magnetic field increases with the increase in the excitation voltage amplitude. When the voltage increases to a certain extent, the material gradually tends to be magnetically saturated. At this time, the magnetic flux density no longer increases with the increase in the magnetic field. If the voltage is increased again, it will only increase the current of the circuit part in the whole device. This will cause an increase in the heat production of components such as resistors, resulting in an increase in the energy consumption of the detection system.

In summary, the frequency affects the detection depth and sensitivity of ENDT. The excitation source amplitude has an important influence on the peak intensity of the magnetic signal. A suitable excitation frequency can realize the effective detection of the sample characteristics within a given depth and cause the detection signal to have a certain intensity, which ensures the sensitivity of ENDT. The appropriate excitation source amplitude can realize the effective separation of interference signals and reduce the energy consumption of the detection system. In the simulation of this paper, the sample to be tested was a 6 mm thick plate sample, and the defect was a through-hole defect. Therefore, the excitation frequency of 10 Hz and the voltage of 25 V could meet the simulation requirements of ENDT.

### 3.3. Variation of Magnetic/Electric Signals with Defects

**Figure 7:**
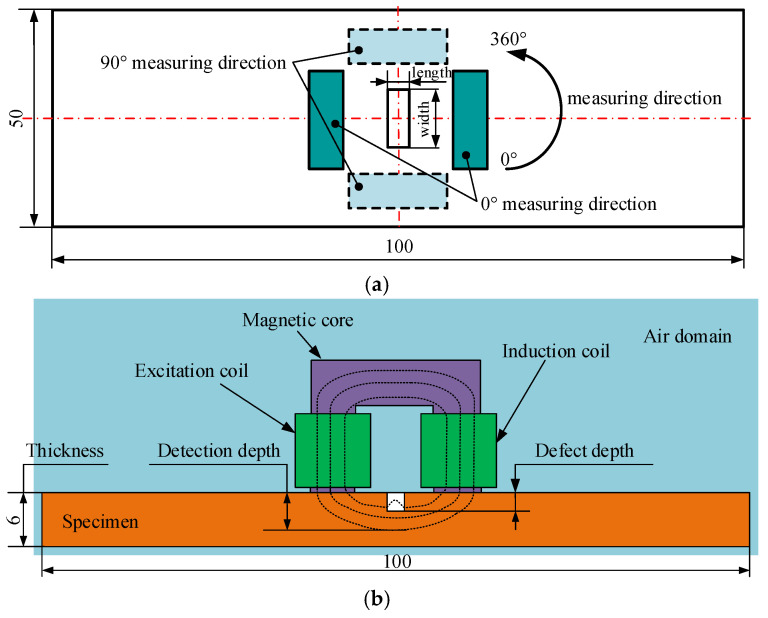
The dimensions of the specimen and the detection angles. (**a**) A schematic of the defect length, defect width, and detection angles. (**b**) A schematic of the detection depth, defect depth, and specimen thickness.

To determine the quantitative evaluation method of ENDT for defect-containing ferromagnetic components, it is necessary to obtain the change law of magnetic/electrical signals with the detection position through finite element simulation. The dimensions of the specimen and the detection position of the magnetic pole relative to the defects during the simulation process are shown in Figure 7.

**Figure 8:**
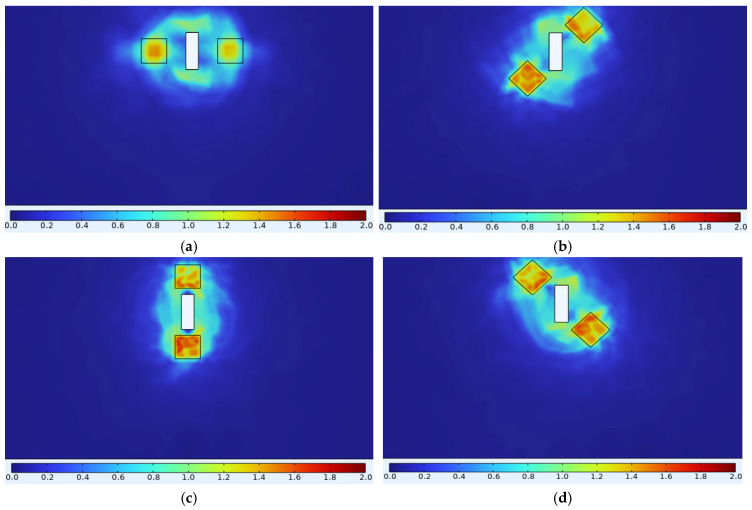

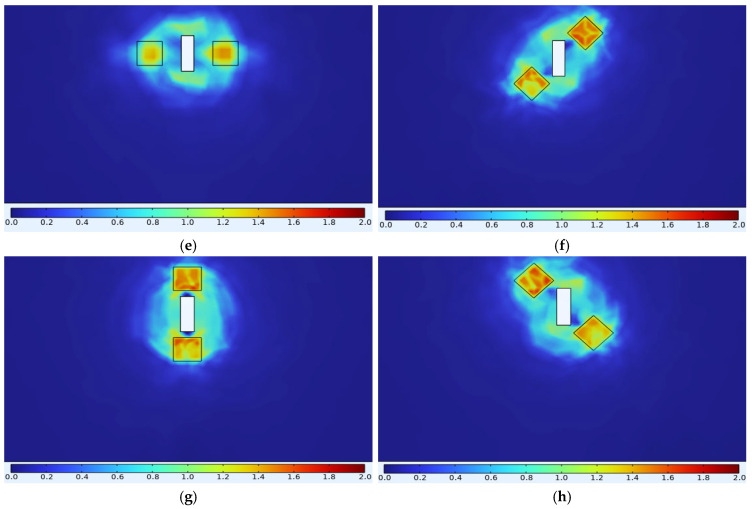
The distributed cloud image of the magnetic flux density signal of the unstressed Q345 specimen containing a defect at different detection positions. (**a**) 0°; (**b**) 45°; (**c**) 90°; (**d**) 135°; (**e**) 180°; (**f**) 225°; (**g**) 270°; (**h**) 315°.

Without considering the influence of hysteresis effects, and with the simulation excitation frequency consistent with the experimental frequency of the B-H curve measurement in Figure 2, the simulation was conducted as follows. After importing the B-H curve shown in Figure 2 into the finite element analysis, the magnetic poles were placed at 0°, 45°, 90°, 135°, 180°, 225°, 270°, and 315° positions, respectively. Subsequently, magnetic flux density signals for an unstressed Q345 specimen containing a defect were obtained through ENDT simulation. Figure 8 illustrates the variations in the strength and distribution of the magnetic flux density peak signals around the defect relative to the detection position, with the contour plot scale expressed in tesla (T). Observations reveal that the magnetic flux density signal strength at the magnetic pole positions significantly exceeds that at other points, and the magnetic flux density strength varies with the orientation of the magnetic poles. Additionally, two centrosymmetric regions of magnetic flux minima exist between the defect edge and adjacent magnetic poles, with distinct semicircular polar regions observed at the 90° and 270° measurement positions. Based on electromagnetic induction principles, the induced electric signals in the detection coil correlate with the perpendicular magnetic flux density component Bz through the bottom surface of the magnetic core. Therefore, this study focuses solely on analyzing Bz and its corresponding induced electrical signals (i.e., electric voltage or electric current) in subsequent simulations.

**Figure 9:**
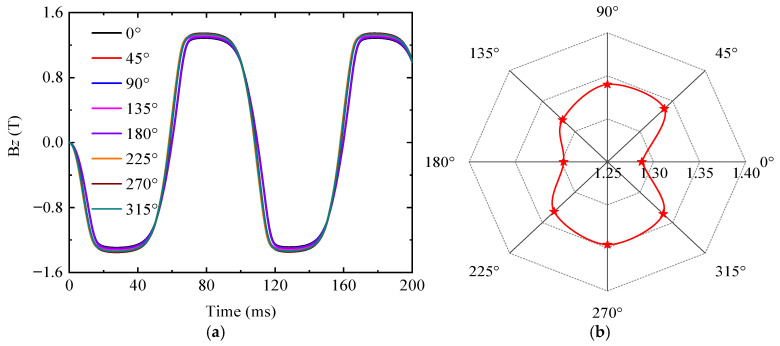
The magnetic flux density signal Bz at different detection positions. (**a**) *Bz*. (**b**) The radar chart of the *Bz* peak value.

To quantitatively analyze the variations in the magnetic flux density (Bz) with the detection position during the defect detection process, Bz signals over two full cycles were obtained and plotted in Figure 9. As shown in Figure 9a, Bz varies periodically with detection position, forming a cucurbit-shaped elliptical pattern with its long axis aligned along the 90–270° axis. Notably, Figure 9b reveals that the Bz peak exhibits extreme values at the 0°, 90°, 180°, and 270° positions, with local minima occurring at the 0° and 180° positions, while local maxima appear at the 90° and 270° positions.

**Figure 10:**
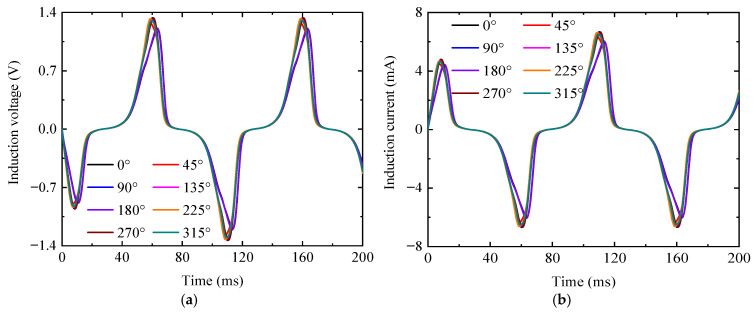
The electric signals of the detection coil at different detection positions. (**a**) The electric voltage signals. (**b**) The electric current signals.

**Figure 11:**
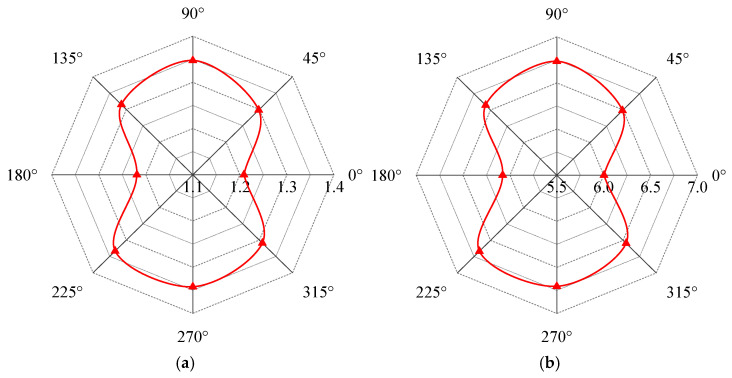
A radar chart of the electric signals’ peak value. (**a**) The electric voltage signals. (**b**) The electric current signals.

Figure 10 presents the electrical signal output from the detection coil across different detection positions. The peak values and occurrence timing of the induced voltage/current signals vary with the detection positions. Combined with Figure 11, the peak and variation pattern of the electrical signals with the detection position are consistent with Figure 9b. Both Figure 9 and Figure 11 demonstrate that the peak curve of Bz and the electrical signal exhibit distinct morphological features, with signal peaks at the 0°, 90°, 180°, and 270° positions defining the characteristic endpoints of these curves. Consequently, the characteristic lengths between these endpoints can serve as quantitative parameters for defect dimensions.

**Figure 12:**
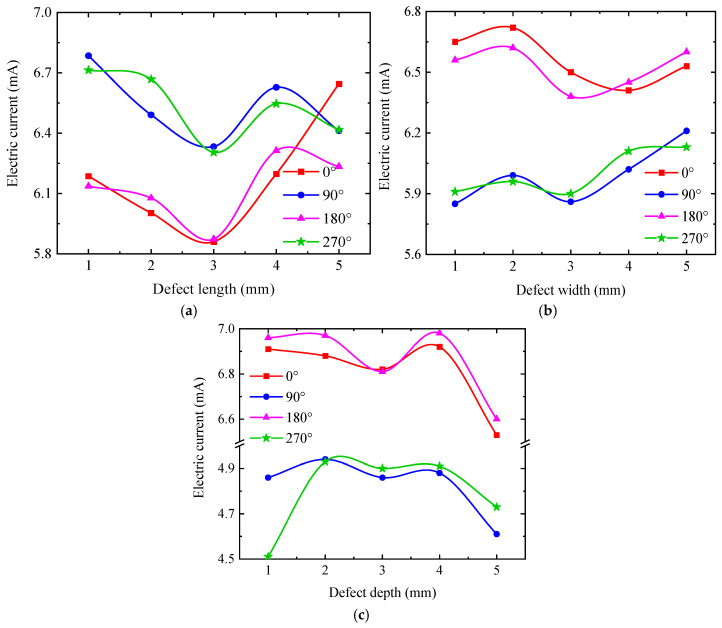
The variation in the detection signal with the defect. (**a**) Defect length. (**b**) Defect width. (**c**) Defect depth.

The distributions of the magnetic flux density signals and peak values of the electrical signals are influenced by defect dimensions. The simulation process was divided into three cases. In Case 1, defect width and depth were fixed at 6 mm, while the lengths varied as follows: 1, 2, 3, 4, and 5 mm. In Case 2, defect length and depth were fixed at 6 mm, while the widths varied as follows: 1, 2, 3, 4, and 5 mm. In Case 3, defect length was fixed at 3 mm and width at 6 mm, while the depths varied as follows: 1, 2, 3, 4, and 5 mm. The variation patterns of the electrical signals with the defect dimensions were analyzed at the 0°, 90°, 180°, and 270° positions. As shown in Figure 12, both magnetic flux density Bz and current signals exhibit irregular variations with defect dimensions. Consequently, the quantitative characterization of defects cannot rely solely on unidirectional detection signals.

**Figure 13:**
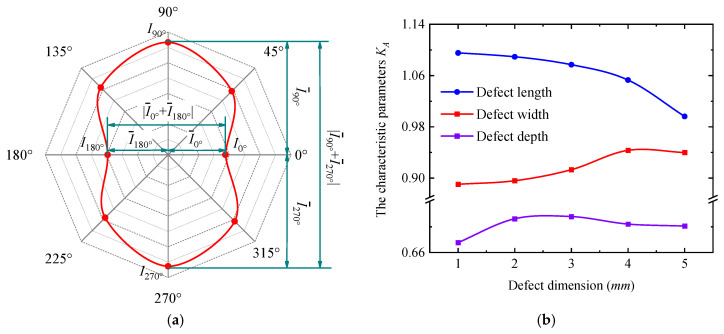
The characteristic parameters of the detection signal. (**a**) The position of each point and the length of the short–long axes of *K_A_* on the signal curve. (**b**) The variation in the characteristic parameters of the peak electrical signal curves with defect dimensions.

Figure 9 and Figure 11 demonstrate that the signals at the 0°, 90°, 180°, and 270° positions correspond to the endpoints of the long/short axes of the peak signal curves. These curves exhibit a distinct elliptical shape (cucurbit shape), thus the peak electrical signals at these four detection positions can be processed as follows:

(13) $$K_{A}=\frac{\left|{\bar{I}}_{90^{\circ }}+{\bar{I}}_{270^{\circ }}\right|}{\left|{\bar{I}}_{0^{\circ }}+{\bar{I}}_{180^{\circ }}\right|}$$

where ${\bar{I}}_{0{}^{\circ}}$, ${\bar{I}}_{90{}^{\circ}}$, ${\bar{I}}_{180{}^{\circ}}$, and ${\bar{I}}_{270{}^{\circ}}$ represent the distance from the peak value of the electrical current signals in the 0°, 90°, 180°, and 270° positions on the signal curve to the origin of the coordinates, respectively. $\left|{\bar{I}}_{0{}^{\circ}}+{\bar{I}}_{180{}^{\circ}}\right|$ is the length between points $I_{0{}^{\circ}}$ and $I_{180{}^{\circ}}$ on the curve, and $\left|{\bar{I}}_{90{}^{\circ}}+{\bar{I}}_{270{}^{\circ}}\right|$ is the length between points $I_{90{}^{\circ}}$ and $I_{270{}^{\circ}}$ on the curve. $K_{A}$ represents the ratio of the long and short axes of the signal curve, and $K_{A}$ is subsequently referred to as the characteristic parameter of the signal curve. The position of each point and the length of the short and long axes are shown schematically in Figure 13a.

According to Equation (13), the *K_A_* parameter quantifies defects by characterizing signal distortion features at different detection positions. Notably, characterizing irregular defect sizes requires detection signals from at least four positions (0°, 90°, 180°, and 270°), whereas for regular rectangular hole defects, only signals from the 0° and 90° positions are sufficient. Compared with conventional magnetic flux leakage detection methods, the newly proposed *K_A_* parameter characterization method significantly reduces the required number of data points.

As shown in Figure 13a, when the characteristic parameter *K_A_* of the peak signal curve exceeds 1, the peak signal curve presents an elliptical morphology, with the long axis along the 90–270° axis. Conversely, when *K_A_* < 1, the long axis shifts to the 0–180° axis. At *K_A_* = 1, the long and short axes of the peak signal curve are equal, resulting in a circular profile. The current signals in Figure 12 were processed using Equation (13), and the derived *K_A_* values are plotted in Figure 13b. As the defect length increases, *K_A_* monotonically decreases from values >1 to values <1, indicating a transition of the long axis from the 90–270° axis to the 0–180° axis. In contrast, *K_A_* increases with defect width, but it increases first and then decreases with defect depth, remaining below 1 in both cases. This confirms that the long axis remains fixed along the 0–180° axis for all depth-related variations. It is noteworthy that the relationship between *K_A_* and the defect size exhibits stronger regularity, thereby facilitating the quantitative evaluation of defects. Critically, the proposed *K_A_* reduces the number of defect detection signals while enhancing detection efficiency.

## 4. Experimental Results and Discussions

Defects within ferromagnetic specimens perturb the distribution of magnetic fields, thereby inducing a variation in detection signals. The three-dimensional characteristics of defects affect the characteristics and strength of the magnetic field, resulting in distinct detection signals across the defect. From Section 3, when the detection magnetic poles are oriented differently with respect to the defect, this can lead to different values of the detection signals. Therefore, the influence of the defect dimension parameters on the detection signals is investigated when the detection magnetic pole is located at the 0°, 45°, 90°, 135°, 180°, 225°, 270°, and 315° positions, and a quantitative relationship between the detection signals and defect dimensions is obtained.

**Figure 14:**
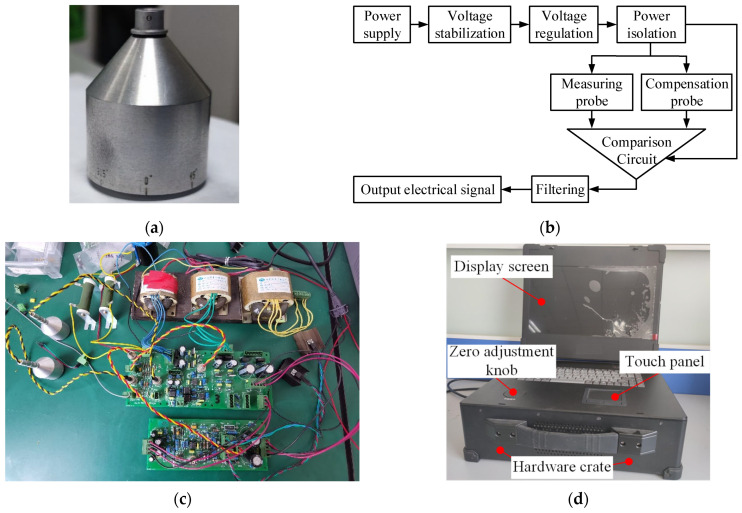
The key structure and integrated system of the ENDT equipment. (**a**) Encapsulated probe. (**b**) The principal schematic of the circuit. (**c**) Part of the hardware circuit. (**d**) The ENDT equipment.

The ENDT equipment used for the defect quantitative evaluation was developed and manufactured by our group in cooperation with Aittest Intelligent Detection Systems (Suzhou, China) Co., as shown in Figure 14. The probe contains a U-shaped magnetic core structure made of a silicon steel material and two coils, with each coil comprising 450 turns of 0.27 mm diameter high-strength enamel-insulated copper wire. After the coil was wrapped around the U-shaped magnetic core, an aluminum shell marked with a scale was used to fix it in position. Subsequently, resin adhesive was injected, and the encapsulation of the probe structure, as shown in Figure 14a, was finalized. The probe was connected to a bridge ratio circuit to ensure equipment sensitivity, and all hardware circuit modules were packaged together. The integrated hardware circuitry was combined with a software system including a computer operating system and detection signal output software, forming the complete ENDT equipment shown in Figure 14d. The detection circuit converts changes in magnetic flux density into changes in the output current of the ENDT system, enabling the derivation of a quantitative relationship curve between the detection signal and defect dimension.

**Figure 15:**
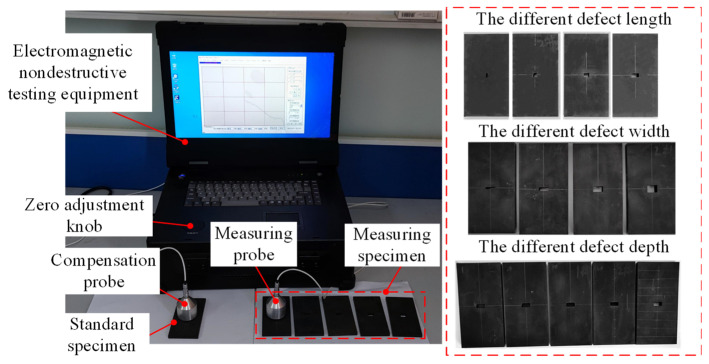
ENDT experimental system for defects.

The schematic diagram of the ENDT experiment for Q345R steel containing defects is shown in Figure 15. The samples used for the experiment were Q345R steel plates with a length of 100 mm, a width of 50 mm, and a thickness of 6 mm. All sample surfaces were smooth and free of scratches, and a rectangular hole defect (in the center of the sample) was machined into each plate. The characteristic dimensions of the defects are listed in Table 4, and their machining accuracy was controlled within ±0.05 mm. After defect preparation, each sample was heated to 500 °C in a muffle furnace at a rate of 20 °C/min, held for 2.5 h, and then furnace-cooled. The above operations effectively reduced the influence of machining stress on the detection results. Finally, the detection surface was marked with a pencil line to ensure accurate positioning of the detection position. The standard samples for the compensation probe shared identical dimensions, machining accuracy, and heat treatment parameters with the detection samples, differing in their defect-free smooth specimens.

During the quantitative defect evaluation ENDT experiment, the equipment was powered on and allowed to warm up for at least 20 min, after which the excitation current was adjusted. The detection probe and compensation probe were aligned in the same detection direction on two smooth, defect-free standard samples of identical specification. A zeroing procedure was then performed to reset the initial current value to zero. Notably, each position was measured at least five times, and the average value (after removing the maximum and minimum values) was recorded as the detection signal for that position. Following the above steps, defects with varying lengths, widths, depths, and detection depths were sequentially analyzed.

### 4.1. The Effect of Defect Length on Detection Signal

**Figure 16:**
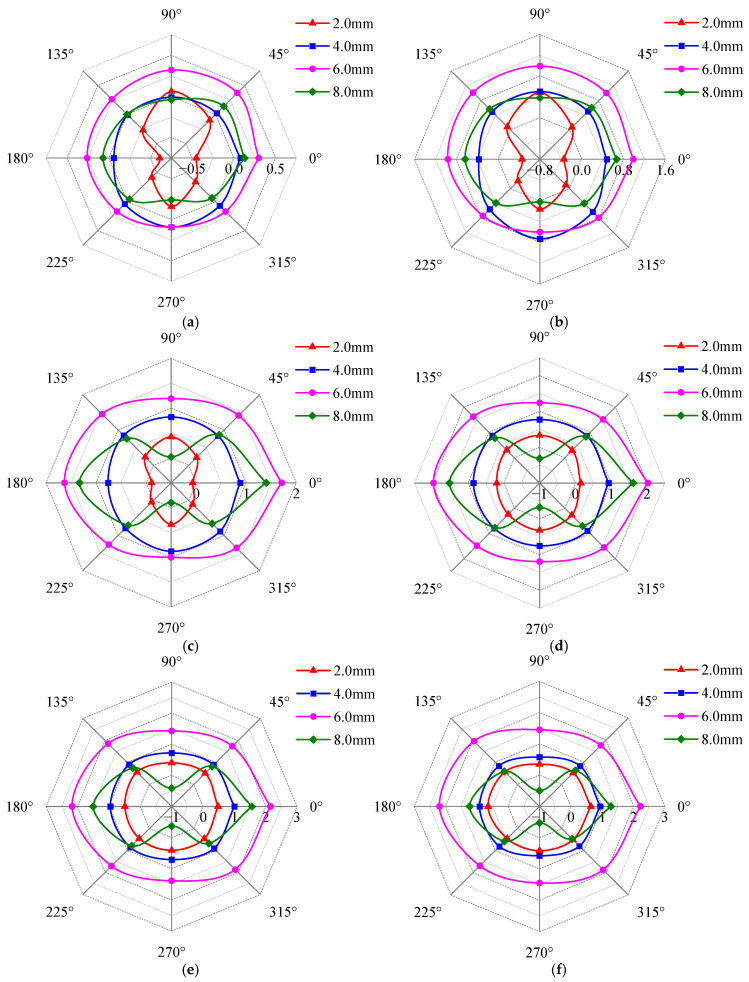
Variation of detection signal with detection position and defect length at different detection depths. (**a**) 0.2 mm; (**b**) 0.5 mm; (**c**) 1.0 mm; (**d**) 1.5 mm; (**e**) 2.0 mm; (**f**) 2.5 mm.

Figure 16 shows the effects of defect length, detection depth, and detection position on the detection current signal, and the unit of the radar plot coordinate axis is mA. From Figure 16, it can be seen that the detection signal at a defect length of 6 mm is larger than that of other defect lengths, which is also the maximum value of the detected signal.

As shown in Figure 16, the detection signal strength gradually increases with detection depth. This occurs because as depth increases, the excitation current rises, enhancing the magnetic field and thereby amplifying the detection signal. When the detection depth exceeds a critical threshold, the excitation current becomes sufficient to drive the material into saturation magnetization. Beyond this point, the detection signal no longer increases with any further excitation current rise. Figure 16 also reveals that the detection signal curves exhibit irregular elliptical shapes (e.g., gourd shape or Cassini egg shape). When the long axis of the signal curve coincides with the 0–180° axis, the local maxima of the detection signal appear at the 0° and 180° positions, while the local minima occur at the 90° and 270° positions. Conversely, when the long axis coincides with the 90–270° axis, the extremum positions exhibit an inverse distribution relative to the 0–180° axis, with the local maxima occurring at the 90° and 270° positions and the local minima observed at the 0° and 180° positions. At the same detection depth (0.2 mm~1.5 mm), as the defect length increases, the long axis of the signal curve changes from the 90–270° axis to the 0–180° axis. For detection depths >1.5 mm, the long axis of all signal curves follows the 0–180° axis, and with the defect length increase, the elliptical shape becomes more obvious. For a same length defect (2 mm~6 mm), the detection signal strength increases with detection depth, and the long axis shifts from the 90–270° axis to the 0–180° axis. Notably, the defect with the 8 mm length always maintains its long axis along the 0–180° axis regardless of depth.

Overall, the signal curve invariably exhibits extreme values at the 0°, 90°, 180°, and 270° positions. Among these, there are two local maxima and two local minima, a phenomenon consistent with the simulation results in Section 3. The effects of defects on detection signals can be explained through two mechanisms. The first explanation is that magnetic circuit reluctance changes. Defects alter the effective cross-sectional area of the magnetic circuit, thereby altering its magnetic circuit reluctance and consequently inducing variations in the inductive current signals of the detection coil. The other explanation is magnetic flux signal changes. Defects partition the specimen into regions of high permeability (ferromagnetic material) and low permeability (air). As magnetic flux lines approach a defect, they deviate to bypass it. This distortion promotes flux leakage into the surrounding air. When the detection probe occupies different positions, defects asymmetrically perturb the magnetic circuit, resulting in the strength of detection signals varying with probe positions.

**Figure 17:**
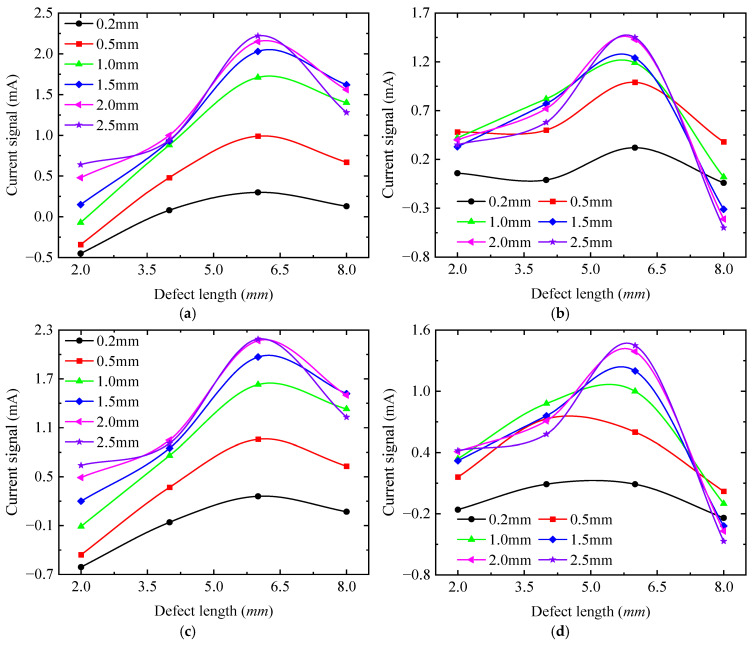
Variation of detection signal with defect length. (**a**) 0° detection position; (**b**) 90° detection position; (**c**) 180° detection position; (**d**) 270° detection position.

**Figure 18:**
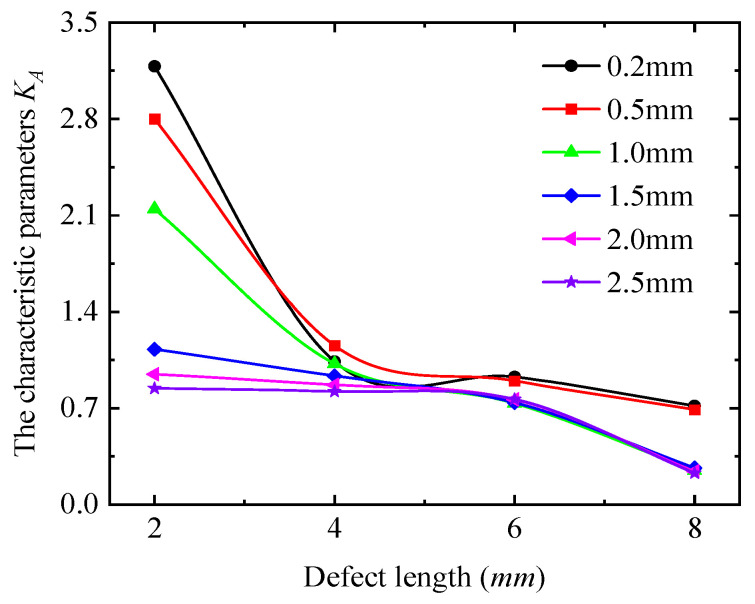
The variation in the characteristic parameters with the defect length.

To describe the quantitative relationship between detection signals and defect length, the detection current signals at the four extreme positions (0°, 90°, 180°, and 270°) were taken for quantitative analysis, as shown in Figure 17. From Figure 17, the detection current signal exhibits a non-monotonic trend, with increasing defect length at all positions, indicating that the detection current signal is not uniquely mapped to defect length. Furthermore, the variation patterns of the signal with defect length differ across positions. These observations confirm that it is not possible to quantitatively analyze the defect dimensions directly from the detection signals. The detection signals at the four extreme positions (0°, 90°, 180°, and 270°) were processed using Equation (13), as shown in Figure 18. The characteristic parameters of the detection signal decrease monotonically with defect length, which is consistent with the simulation results in Section 3. For detection depths < 1.5 mm, the characteristic parameters decrease from a constant of >1 to a constant of <1, indicating that the long axis changes from the 90–270° axis to the 0–180° axis. For detection depths > 2 mm, the characteristic parameters remain <1, confirming that all signal curves are ellipses with long axes fixed along the 0–180° direction.

### 4.2. The Effect of Defect Width on Detection Signal

**Figure 19:**
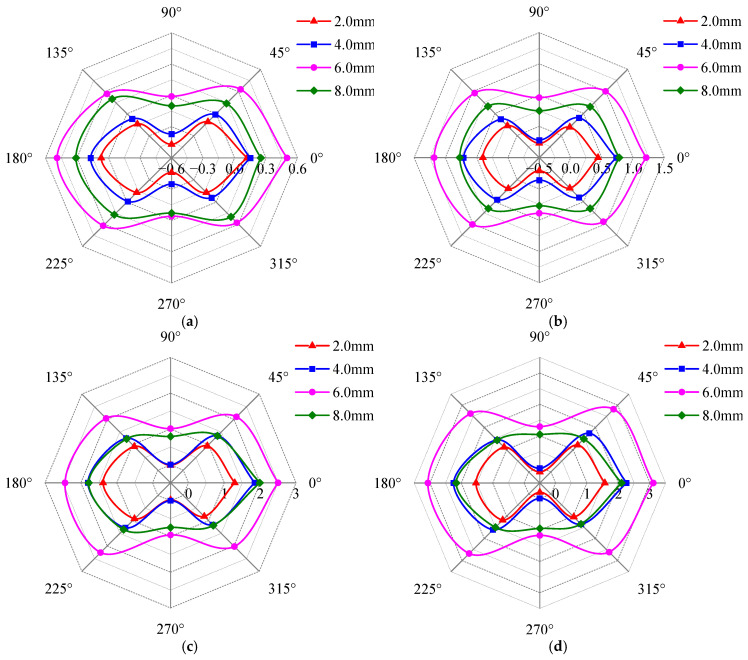

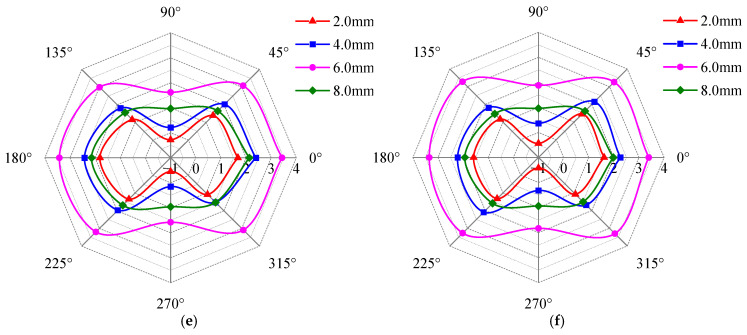
Variation in detection signal with detection position and defect width at different detection depths. (**a**) 0.2 mm; (**b**) 0.5 mm; (**c**) 1.0 mm; (**d**) 1.5 mm; (**e**) 2.0 mm; (**f**) 2.5 mm.

Figure 19 illustrates the effects of defect width, detection depth, and detection position on the detection current signal, and the unit of the radar plot coordinate axis is mA. As shown in Figure 19, at each detection position, the detection current signal increases with detection depth, but its growth rate gradually diminishes. Within the 2 mm~2.5 mm detection depth range, the signal stabilizes, with negligible further increase, and these changes are consistent with Section 4.1. All signal curves maintain elliptical shapes with long axes aligned along the 0–180° axis, where local maxima consistently occur at the 0° and 180° positions, and local minima at the 90° and 270° positions. This confirms that defect width variations do not alter the long-axis orientation or extremum positions. Under a constant detection depth, an increase in defect width reduces the relative difference between the maximum and minimum values, thereby inducing a convergence of the elliptical curve toward circularity.

**Figure 20:**
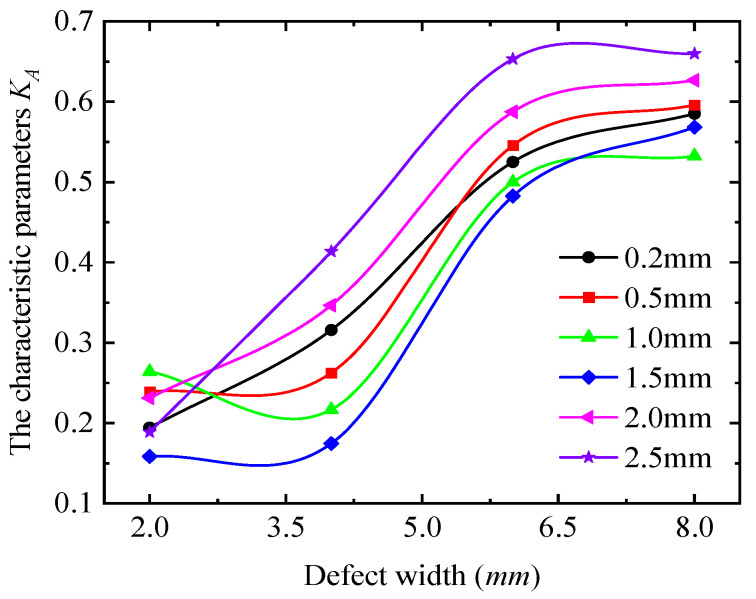
The variation in the characteristic parameters with the defect width.

Figure 19 further reveals that the detection signal strength for a 6 mm defect width exceeds that of other defect widths. While the signal strength varies with detection depth for a given width, the curve shapes remain nearly identical across depths. To quantify the relationship between detection signals and defect width, the current signals at the four extreme value positions (0°, 90°, 180°, and 270°) were processed using Equation (13), as shown in Figure 20.

As shown in Figure 20, the characteristic parameters of the signal curve increase with defect width and approach a value of 1, yet their maximum values remain below 0.7. This indicates that while a defect alters the characteristic parameters, it does not affect the positions of the long and short axes of the signal curve. Specifically, as the defect width increases, the characteristic parameters asymptotically approach 0.7, implying that the short-axis length gradually converges toward the long-axis length, thereby causing the signal curve to become circular.

### 4.3. The Effect of Defect Depth on Detection Signal

**Figure 21:**
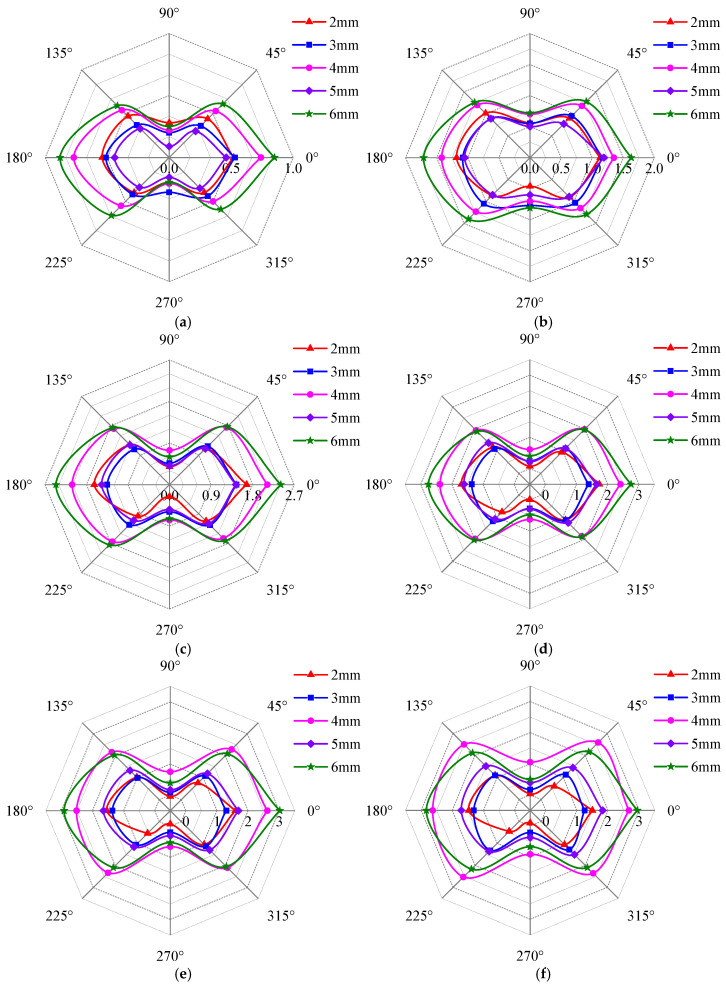

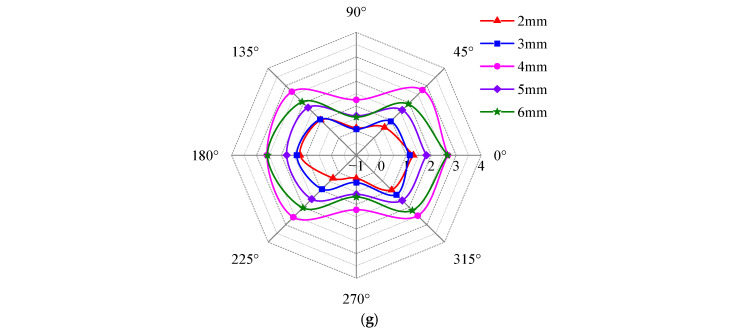
Variation in detection signal with detection position and defect depth at different detection depths. (**a**) 0.2 mm; (**b**) 0.5 mm; (**c**) 1.0 mm; (**d**) 1.5 mm; (**e**) 2.0 mm; (**f**) 2.5 mm; (**g**) 3 mm.

To investigate the effects of defect depth on detection signals, rectangular blind holes and through holes with varying depths were machined at the center of Q345R samples. The hole fillet radius was set to *R* = 1 mm, and the defect dimensions are listed in Table 4. Figure 21 illustrates the variation in the detection current signal with the detection depth, the detection position, and defect depth within 0.2 mm~3 mm, and the unit of the radar plot coordinate axis is mA. Figure 21 shows that the envelope area of the signal peak curve is the largest at a defect depth of 6 mm. The detection signal strength increases with detection depth, while the elliptical curve shape (long axis along the 0–180° axis) remains consistent with Figure 19. The local maxima value occurs at the 0° and 180° positions, and the local minima at the 90° and 270° positions, confirming that the defect depth does not alter the long-axis orientation or extremum positions of the signal curve. Notably, within the 0.2 mm~1.5 mm detection depth range, defect depths exceed detection depths. Here, signal variations correlate with defect depth, suggesting that surface-layer signals can evaluate sub-surface defects. At 2 mm~3 mm detection depths, defects with 2 mm depth are fully enveloped by the skin-effect magnetic field, inducing curve distortion distinct from the 0.2 mm to 1.5 mm range. Overall, the strength and curve shape of the detection signal are affected by the depth defect, which indicates that the detection signal at the corresponding depth can realize the effective evaluation of the defect depth.

**Figure 22:**
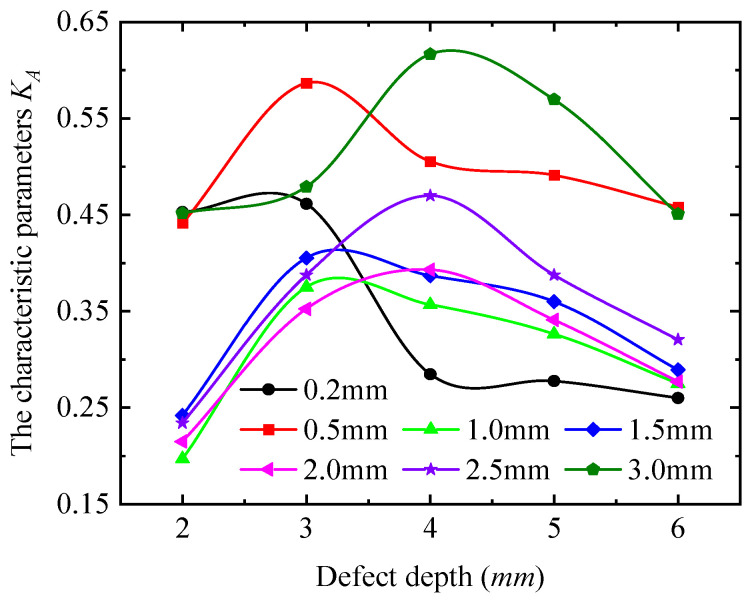
The variation in the characteristic parameters with the defect depth.

To quantitatively analyze the variation pattern of detection signals with defect depth, the signals at the four extreme points (0°, 90°, 180°, and 270°) were processed using Equation (13), and the results are shown in Figure 22. The characteristic parameters increase first and then decrease with increasing defect depth, and their values remain below 0.65. This indicates that the positions of the long/short axes of the signal curve are unaffected by changes in defect depth. Remarkably, the slopes of the characteristic parameter curves for detection signals vary with defect depth, and each defect size corresponds to a unique slope value. This distinct slope characteristic can serve as a basis for defect quantification. Additionally, as the detection depth increases, the defect depth corresponding to the turning point of the characteristic parameters gradually increases. Notably, the trend in characteristic parameters with defect depth differs from that observed for defect length and width.

In summary, the ENDT signal curves for defects are elliptical. The signal strength increases with detection depth and eventually stabilizes. The characteristic parameter of the signal curve decreases from a constant of >1 to a constant of <1 as the defect length increases, indicating that defect length alters the long-axis position of the signal curve. The characteristic parameters increase with defect width, but first increase and then decrease with defect depth. However, all characteristic parameters for defect width and depth remain below 1, suggesting that changes in these dimensions do not affect the long-axis position of the curve.

## 5. Conclusions

Defects generated during long-term service are a primary cause of failure in ferromagnetic components. The ability to accurately detect defects in ferromagnetic materials is critical for component safety assessment. This paper investigates ENDT for ferromagnetic material defects, with the following conclusions:

(1) Based on the electromagnetic field theory and ENDT characteristics, the effects of excitation frequency and voltage amplitude on detection signals were studied. The results show that excitation frequency affects ENDT detection depth and sensitivity, while voltage amplitude influences magnetic signal peak strength. Selecting an appropriate voltage amplitude enables the effective separation of interference signals. The excitation frequency and amplitude were determined as 10 Hz and 25 V, respectively.

(2) The influence of regular rectangular defect dimensions on ENDT signals was analyzed. The results demonstrate that the peak curves of Bz and the electrical signal are elliptical, with the local extrema occurring at the 0°, 90°, 180°, and 270° positions. The characteristic parameter *K_A_* of the electrical signal peak curve is proposed as a quantitative evaluation index of defects. Notably, calculating *K_A_* for regular defects requires only two detection signals with 90° separation, whereas irregular defects necessitate at least four signals with 90° intervals.

(3) Quantitative experiments reveal that *K_A_* decreases (from a constant of >1 to a constant of <1) with increasing defect length, increases with defect width, and first increases and then decreases with defect depth. However, *K_A_* values for defect width and depth remain below 1. A method using *K_A_* to evaluate ferromagnetic material defects is proposed, revealing the relationship between *K_A_* and defect dimensions. Finally, a novel ENDT evaluation method for defects is formed.

This study combines theoretical analysis, simulation analysis, and quantitative experiments to investigate ENDT for ferromagnetic material defects. A simulation model, signal processing method, and a novel ENDT evaluation method are developed. Compared to the existing methods, the method proposed in this paper requires fewer detection points while maintaining accuracy, simplifying defect pattern identification. These advancements enhance ENDT precision and promote its engineering applications.

## Figures and Tables

**Table 1:** Chemical composition of Q345R (wt%).

Elements	C	Si	Mn	P	S	Nb	V	Ti	Cr	Ni	Cu	Mo
Percentage	0.17	0.42	1.5	0.014	0.009	0.002	0.002	0.001	0.043	0.008	0.01	0.008

**Table 2:** The parameters of the simulation model.

Turn Number	Cross-Sectional Area of Coil Wire (m^2^)	Sample Conductivity (S/m)	Coil Conductivity (S/m)	Circuit Resistance R1 (Ω)	Circuit Resistance R2 (Ω)
450	1 × 10^−6^	1.2 × 10^7^	6 × 10^7^	200	200

**Table 3:** The parameters of the J-A model.

Parameter	Saturation Magnetization (A/m)	Domain Wall Density (A/m)	Pinning Loss (A/m)	Magnetization Reversibility	Inter-Domain Coupling
Values on the diagonal	1.31 × 10^6^	233.78	374.96	0.74	5.62 × 10^−4^
	1.33 × 10^6^	177.86	232.65	0.65	4.17 × 10^−4^
	1.31 × 10^6^	233.78	374.98	0.74	5.62 × 10^−4^

**Table 4:** The dimensions of the preparation of defects.

Defect Size	Length (mm)	Width (mm)	Depth (mm)
length experiment	2, 4, 6, 8	10	6
width experiment	5	2, 4, 6, 8	6
depth experiment	5	10	2, 3, 4, 5, 6

## Data Availability

All data generated or analyzed during this study are included in this published article.
